# Unique inhibitory cascade pattern of molars in canids contributing to their potential to evolutionary plasticity of diet

**DOI:** 10.1002/ece3.436

**Published:** 2013-01-03

**Authors:** Masakazu Asahara

**Affiliations:** Graduate School of Sciences, Kyoto UniversityKyoto, 606-8502, Japan

**Keywords:** Canidae, Carnivora, dental formulae, dental morphology, evolvability, inhibitory cascade

## Abstract

Developmental origins that guide the evolution of dental morphology and dental formulae are fundamental subjects in mammalian evolution. In a previous study, a developmental model termed the inhibitory cascade model was established. This model could explain variations in relative molar sizes and loss of the lower third molars, which sometimes reflect diet, in murine rodents and other mammals. Here, I investigated the pattern of relative molar sizes (inhibitory cascade pattern) in canids, a taxon exhibiting a wide range of dietary habits. I found that interspecific variation in canid molars suggests a unique inhibitory cascade pattern that differs from that in murine rodents and other previously reported mammals, and that this variation reflects dietary habits. This unique variability in molars was also observed in individual variation in canid species. According to these observations, canid species have greater variability in the relative sizes of first molars (carnassials), which are functionally important for dietary adaptation in the Carnivora. In conclusion, an inhibitory cascade that differs from that in murine rodents and other mammals may have contributed to diverse dietary patterns and to their parallel evolution in canids.

## Introduction

Dental morphology and dental formulae are important taxonomic traits in mammals (Ungar 2010), and are also used for paleoecological and ecomorphological studies in mammals because these traits reflect dietary adaptations (Popowics 2003; Benton 2004; Friscia et al. 2007; Van Valkenburgh 2007). Patterns of adaptation are guided by the variability and evolvability of these traits (Klingenberg 2005; Barton et al. 2007). In fact, evolvability, that is, systems having variability, generating new variation (Wagner and Altenberg 1996; Kirschner and Gerhart 1998), is fundamental to the evolution of traits (Klingenberg 2005; Futuyma 2010). Therefore, developmental mechanisms that guide and constrain patterns of adaptation in dental morphology and dental formulae are crucial subjects for elucidating their proximate and ultimate factors, and the interactions between these factors during dental evolution (Kavanagh et al. 2007; Laland et al. 2011; Wilson 2011). Therefore, many recent studies have focused on the developmental mechanism of the evolution of dental morphology and dental formulae, which guide and constrain the reflection of dietary adaptations (e.g. Kavanagh et al. 2007; Polly 2007; Laffont et al. 2009; Renvoisé et al. 2009; Harjunmaa et al. 2012; Wilson et al. 2012). A recent developmental study established a developmental model that can explain evolution of the relative sizes of lower molars in murine rodents (Kavanagh et al. 2007). This model, termed the inhibitory cascade model, explains the relative sizes of the lower molars (first, second, and third molars; M_1_, M_2_, and M_3_, respectively) by the balance of inhibitor molecules from M_1_ tooth germ and activator molecules from mesenchyme during dental development. Inhibitor molecules inhibit the development of distal molars, whereas activator molecules activate their development. For example, greater inhibition generates a larger M_1_ and smaller M_3_ (M_1_ >> M_2_ >> M_3_), while lower inhibition and greater activation generate equal-sized molars (M_1_ = M_2_ = M_3_), and moderate inhibition and activation generate an intermediate condition (M_1_ > M_2_ > M_3_) (Kavanagh et al. 2007). This model can explain dental variations that have resulted from dietary adaptations in murine rodents (Kavanagh et al. 2007). Faunivorous murine species exhibit M_1_ >> M_2_, and have lost M_3_. Conversely, herbivorous murine species have approximately equal-sized molars. For most mammals, from marsupials to various placental orders, the relative sizes of M_1_, M_2_, and M_3_ change sequentially and thus were explained by the model (Polly 2007). Several authors have investigated relative molar sizes in several mammalian taxa and have reported differences in the inhibitory cascade pattern between a number of taxa including murine rodents (Renvoisé et al. 2009; Wilson et al. 2012). It has been noted that the variability of a trait initiates the evolvability of that trait (Klingenberg 2005; Barton et al. 2007; Wilson 2011). It is possible that the unique patterns of inhibitory cascade that guide variability in a particular taxon could promote the evolvability of typical molar patterns and, consequently, the evolvability of diet in that taxon.

The order Carnivora, and particularly the family Canidae (canids), is one of the most diverse mammalian taxa in terms of dietary pattern, and exhibits parallel evolution in diet (Van Valkenburgh and Koepfli 1993; Friscia et al. 2007; Goswami 2010; Sillero-Zubiri 2010). Similar to faunivorous murine rodents, several canid species have lost M_3_ (Sillero-Zubiri 2010); this loss is thought to be related to the enlargement of carnassial teeth (M_1_) – a carnivorous adaptation for shearing flesh – and to the degeneration of molars M_2_ and M_3_ (Holliday 2010). However, patterns of relative molar sizes and dietary adaptation in canids, and the relationship of these parameters to the inhibitory cascade model are still not clear. In this study, my primary objective was to elucidate patterns of interspecific variation in the relative sizes of lower molars in canids, and to determine the relationship of this variation to the inhibitory cascade model and to dietary adaptations.

A second objective was to elucidate the variability within the species in canids that guides evolutionary patterns (Klingenberg 2005). To achieve these objectives, I investigated individual variation in relative molar sizes as an indication of variability in this parameter at the intraspecific level (Klingenberg 2005). In addition, I investigated individual variation in the number of teeth, as oligodonty (missing teeth) is considered a transitional stage in the evolution of dental formulae (e.g. Ohtaishi 1986; Giannini and Simmons 2007). An earlier experimental study examining mouse development found that the number of molars was affected by the inhibitory cascade (Kavanagh et al. 2007). Therefore, I also compared individual variation in relative molar sizes and number of molars (i.e. congenital missing of M_3_), to consider the inhibitory cascade and the evolutionary process of M_3_ loss in canids.

## Material and Methods

I examined 320 specimens from 27 species of canids (Canidae, Carnivora, Mammalia) (Table 1). All species were examined to clarify evolutionary patterns in relative molar sizes. The dietary pattern of each species was categorized as carnivorous (primarily eating mammalian flesh), omnivorous (eating various foods, with neither mammalian flesh nor insects comprising >50% of the diet), or insectivorous (primarily eating insects) using information from the literature (Sillero-Zubiri 2010). In order to estimate variability in molars, I examined individual variation in relative molar sizes. For this purpose, individual variation within seven species was examined whereby I measured >15 individuals from each species (Table 2). In addition, I examined individual variation in the presence or absence of M_3_ in *Vulpes lagopus* and *Nyctereutes procyonoides* to clarify whether individual variation and missing of M_3_ are explained by the inhibitory cascade model. The specimens of *N. procyonoides* examined were those deposited in Kyoto University Museum, Kyoto University, Japan, which had been collected from a small island, Chiburi Island, Shimane Prefecture, Japan. Specimens of the other species were those deposited in the Department of Mammalogy, American Museum of Natural History, USA, which had been collected from large areas. I measured the size of each molar as the projected area in photos taken from the occlusal view using ImageJ software (NIH, Bethesda, MD), and compared the relative molar sizes in the morphospace: M_2_ size/M_1_ size versus M_3_ size/M_1_ size (abbreviated as M_2_/M_1_ vs. M_3_/M_1_) (Kavanagh et al. 2007). Any given point in morphospace represents the relative sizes of the three molars of a particular species or individual. I plotted the average values for each species to describe interspecific variation, and plotted each individual to describe individual variation. Reduced major axis (RMA) regressions were performed on these plots after performing Anderson-Darling normality test. I used M_2_/M_1_ as an index of activation versus inhibition during molar development. M_2_/M_1_ scores between carnivorous and omnivorous species were compared using the Mann–Whitney *U* test. Further, for *V. lagopus* and *N. procyonoides*, M_2_/M_1_ scores were compared between normal individuals and individuals that were missing M_3_ on one or both sides. Statistical analyses were performed using Minitab 14 (Minitab, Inc., PA), and RMA regressions were performed using PAST (Hammer et al. 2001). Several studies have utilized multiple regressions to elucidate how absolute molar sizes affect one another (Renvoisé et al. 2009; Wilson et al. 2012). However, this method tends to reflect variability in the absolute size of M_1_, and activation versus inhibition patterns can become obscured. Therefore, I focused on relative molar sizes, that is, the inhibitory cascade.

**Table 1:** Species examined in this study, and their molar ratios

Number	Species	Diet	*N*	M2/M1 ± SD	M3/M1 ± SD
1	*Atelocynus microtis*	Omnivorous	3	0.54 ± 0.04	0.15 ± 0.04
2	*Canis adustus*	Omnivorous	4	0.51 ± 0.12	0.17 ± 0.05
3	*Canis aureus*	Omnivorous	11	0.42 ± 0.04	0.11 ± 0.02
4	*Canis latrans*	Carnivorous	51	0.37 ± 0.03	0.09 ± 0.02
5	*Canis lupus*	Carnivorous	28	0.31 ± 0.03	0.09 ± 0.01
6	*Canis mesomelas*	Omnivorous	20	0.36 ± 0.02	0.10 ± 0.02
7	*Cerdocyon thous*	Omnivorous	5	0.54 ± 0.06	0.16 ± 0.03
8	*Chrysocyon brachyurus*	Omnivorous	3	0.46 ± 0.01	0.18 ± 0.01
9	*Cuon alpinus*	Carnivorous	2	0.25 ± 0.00	0.00 ± 0.00
10	*Lycalopex culpaeus*	Omnivorous	10	0.41 ± 0.03	0.11 ± 0.02
11	*Lycalopex griseus*	Omnivorous	15	0.50 ± 0.05	0.13 ± 0.02
12	*Lycalopex gymnocercus*	Omnivorous	9	0.53 ± 0.02	0.13 ± 0.01
13	*Lycalopex sechurae*	Omnivorous	5	0.53 ± 0.04	0.16 ± 0.01
14	*Lycalopex vetulus*	Insectivorous	6	0.71 ± 0.12	0.25 ± 0.11
15	*Lycaon pictus*	Carnivorous	7	0.32 ± 0.02	0.07 ± 0.02
16	*Nyctereutes procyonoides*	Omnivorous	44	0.47 ± 0.03	0.07 ± 0.04
17	*Otocyon megalotis*	Insectivorous	7	0.97 ± 0.05	0.82 ± 0.06
18	*Speothos venaticus*	Carnivorous	4	0.17 ± 0.04	0.00 ± 0.00
19	*Urocyon cinereoargenteus*	Omnivorous	31	0.53 ± 0.03	0.14 ± 0.03
20	*Vulpes bengalensis*	Omnivorous	2	0.61 ± 0.03	0.23 ± 0.04
21	*Vulpes chama*	Omnivorous	1	0.62 ± 0.00	0.20 ± 0.00
22	*Vulpes lagopus*	Carnivorous	31	0.32 ± 0.04	0.07 ± 0.04
23	*Vulpes macrotis*	Omnivorous	4	0.39 ± 0.05	0.10 ± 0.00
24	*Vulpes pallida*	Omnivorous	1	0.72 ± 0.00	0.23 ± 0.00
25	*Vulpes velox*	Omnivorous	7	0.39 ± 0.01	0.09 ± 0.01
26	*Vulpes vulpes*	Carnivorous	3	0.35 ± 0.01	0.10 ± 0.00
27	*Vulpes zerda*	Omnivorous	6	0.58 ± 0.04	0.16 ± 0.03

**Table 2:** Regression results (RMA) of the M_2_/M_1_ versus M_3_/M_1_ morphospace, showing confidence intervals (CI)

	Types of variation	Slope	CI max	CI min	Intercept	CI max	CI min	*r*	*P*	*N*
Inhibitory cascade model		2.00			−1.00					
Canidae (with 3 molars)	Interspecific	0.45	0.515	0.376	−0.08	−0.037	−0.104	0.91	0.000	24
Canidae (without *O. megalotis*)	Interspecific	0.48	0.537	0.412	−0.09	−0.057	−0.119	0.93	0.000	26
Canidae (on diet)	Diet	0.48	0.538	0.438	−0.09	−0.061	−0.111	0.99	0.035	3
*Canis latrans*	Individual	0.52	0.632	0.380	−0.10	−0.041	−0.138	0.41	0.003	51
*Canis lupus*	Individual	0.49	0.606	0.267	−0.07	0.003	−0.101	0.69	0.000	28
*Canis mesomelas*	Individual	0.71	2.059	0.240	−0.16	0.011	−0.649	0.43	0.060	20
*Lycalopex griseus*	Individual	0.46	0.594	0.184	−0.09	0.036	−0.163	0.60	0.018	15
*Urocyon cinereoargenteus*	Individual	0.94	1.150	0.676	−0.36	−0.220	−0.466	0.54	0.002	31
*Vulpes lagopus* (with 3 molars)	Individual	0.73	0.946	0.425	−0.16	−0.052	−0.233	0.78	0.001	15
*Nyctereutes procyonoides* (with 3 molars)	Individual (small island)	0.82	2.096	0.560	−0.32	−0.186	−0.984	0.55	0.036	26

## Results

**Figure 1:**
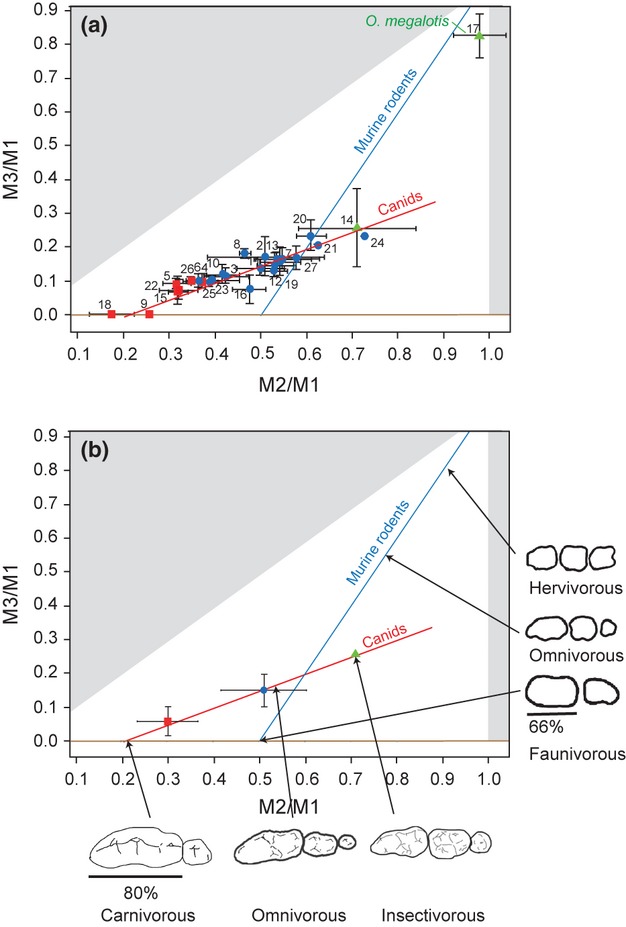
Variation in relative molar sizes among canid species. Interspecific variation in canids, excluding *Otocyon megalotis* (red line), differed from that in murine rodents (blue line). Colors and shapes indicate diet of a given species (red square: carnivorous, blue circle: omnivorous, green triangle: insectivorous) (Table 1). (a) Species plots with standard deviations (SD). Numbers indicate species as in Table 1. (b) Dietary patterns with SD. Occlusal view of molar rows of species for each diet in canids and murine rodents. Illustration of murine rodents after Kavanagh et al. (2007). Maximum relative M_1_ size reaches 80% of the total molar row in canids, and 66% in murine rodents.

As a result of interspecific variation, plots of the molar ratios of all species in morphospace indicated that relative molar sizes changed sequentially (i.e. M_1_ > M_2_ > M_3_; plots are in the white zone in Fig. 1). These results are in agreement with the consensus area of the inhibitory cascade model suggested by Polly (2007). Interspecific variation in relative molar sizes among the majority of canid species, excluding *Otocyon megalotis*, exhibited a pattern that differed in slope from the variation observed in murine rodents (Kavanagh et al. 2007). That is, the area in which *O. megalotis* was plotted indicated that it had similar sized molars (Fig. 1). Including *O. megalotis* data in M_3_/M_1_ scores caused the assumption of normality to be violated; therefore, this species was excluded from interspecific regression analysis. The pattern of interspecific variation revealed a correlation between M_2_/M_1_ and M_3_/M_1_ (*P* < 0.001; Table 2). The slope of the variation among murine rodents was higher than, and outside of the 95% confidence interval of, that of canids (Table 2), indicating that the cascade patterns of canids and murine rodents are significantly different (Fig. 1, Table 2). According to this pattern, the relative sizes of M_1_ in canids varied greatly in relation to that of murine rodents. Consequently, the maximum relative size of M_1_ occupied 80% of the total molar row in canids, but only 66% of that in murine rodents (Fig. 1). Carnivorous species tended to have lower M_2_/M_1_ scores than omnivorous species (two-sided *U* test, *W* = 21.0, *P* < 0.001). That is, some carnivorous species have much larger M_1_ and smaller M_2_ and M_3_, than omnivorous species. Notably, species at the extremes of the distribution, that is, *Cuon alpinus* and *Speothos venaticus*, have lost M_3_. These differences in relative molar sizes and loss of M_3_ have evolved in parallel within many clades of canids (Fig. 3).

**Figure 2:**
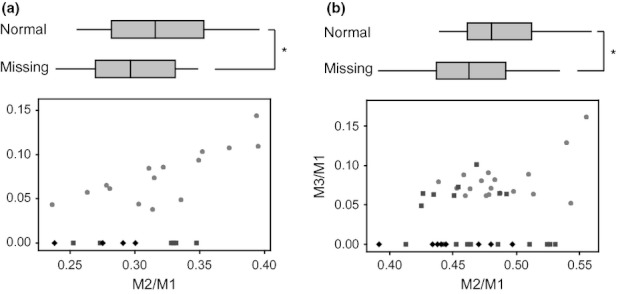
Individual variation in relative molar sizes in (a) *Vulpes lagopus* and (b) *Nyctereutes procyonoides,* indicating presence or absence of M_3_ by color (black: missing on both sides; dark gray: missing on one side; right gray: normal). Within species, M_2_/M_1_ scores differed significantly between normal individuals and individuals missing M_3_ (indicated by asterisks).

As the result of individual variation, normality of the M_3_/M_1_ data was not observed for *V. lagopus* and *N. procyonoides*, species in which individuals were missing M_3_. When individuals with dental anomalies were excluded, the data for all species were normally distributed (*P* < 0.05), and regression analyses were performed. In the M_2_/M_1_ versus M_3_/M_1_ morphospace, individual variations were correlated in most of the species (*P* < 0.05; Table 2), with the exception of *Canis mesomelas* (*P* = 0.06; Table 2). That is, individuals with relatively larger M_1_ tended to have relatively smaller M_2_ and M_3_, and vice versa (Fig. 2). Ten individuals of *V. lagopus* and 25 individuals of *N. procyonoides* were missing M_3_ on one or both sides (32% and 56%, respectively). There was no evidence of concrescence, and all cases of missing teeth were considered to be congenital. In both *V. lagopus* and *N. procyonoides*, individuals missing one or two M_3_ had lower scores for M_2_/M_1_ (i.e. greater inhibition) than normal individuals (one-sided *U* test, *W* = 169.0 and 509.0, respectively, *P* < 0.05) (Fig. 2). Individuals with relatively larger M_1_ and smaller M_2_ tended to be missing M_3_.

## Discussion

### The inhibitory cascade pattern in canids and its relationship to diet

Sequential changes in relative molar sizes (i.e. M_1_ > M_2_ > M_3_; plots are in the white zone in Fig. 1) and correlation between M_2_/M_1_ and M_3_/M_1_ have been considered as evidence that relative molar sizes are regulated by an inhibitory cascade, indicating that there are single mechanisms that inhibit distal molars (Kavanagh et al. 2007; Polly 2007). Therefore, variation in relative molar sizes in canid species is also regulated by an inhibitory cascade. In this study, carnivorous species tended to have lower M_2_/M_1_ scores (or relatively larger M_1_) than omnivorous species. Moreover, the two carnivorous species having the smallest M_2_ in relation to M_1_ have lost M_3_. Carnivorous species exhibited the pattern M_1_ >> M_2_ >> M_3_, but omnivorous species exhibited M_1_ > M_2_ > M_3_. As the number of insectivorous species was limited, they could not be analyzed statistically; however, these species tended to have more equal-sized molars. Thus, the inhibitory cascade reflects dietary adaptation in canid molars (Fig. 1). The relationships among inhibitory cascade, relative molar sizes, and dietary adaptation in canids are similar to those in murine rodents (Kavanagh et al. 2007). However, the patterns of adaptation differ between canids and murine rodents. For example, insectivorous canids and herbivorous murines have equal-sized molars, and carnivorous canids and faunivorous murines (eating animals including insects) have relatively larger M_1_. This may be due to the difference in absolute body size among the species. Insects are sufficiently large prey for murines, and these mammals need to concentrate their masticatory function on one major tooth. Similarly, mammalian flesh is sufficiently large to be accommodated by canid molars. In contrast, insects are small food items for canids, and canids require a long molar row with equal-sized teeth in order to chew a number of insects at once (Ungar 2010).

Among the species examined, only *O. megalotis* had equal-sized molars, and was located distantly from the other canids in morphospace (Fig. 1). This may be related to the unique characteristic in *O. megalotis* of having four lower molars (Sillero-Zubiri 2010; Ungar 2010). Interspecific variation in the other canids exhibited a unique pattern of molar ratios that differs from that in murine rodents (Kavanagh et al. 2007), indicating a difference in the inhibitory cascade. Such differences have been reported in previous studies; however, the slope of the difference in canids was lower than that in any previously reported taxa (canids, interspecific: 0.48; canids, individual: 0.46–0.94; murine and arvicoline rodents and South American ungulates: 1.17–2.15) (Kavanagh et al. 2007; Renvoisé et al. 2009; Wilson et al. 2012). In mouse experiments, all inhibition molecules were eliminated and interspecific variation in murine rodents was identical to observed variation in molar proportions (Kavanagh et al. 2007). However, diffusion patterns may differ between inhibition molecules. It is possible that particular molecules with low diffusion efficiency have high evolvability and generate unique slopes in canids; however this is not yet clear.

### Variability in relative molar sizes and loss of M3 in canids

The results of individual variation clearly showed the correlations between M_2_/M_1_ and M_3_/M_1_ (Table 2), and different M_2_/M_1_ scores between individuals having M_3_ vs. individuals in which M_3_ was missing (Fig. 2), indicating that greater inhibition results in smaller distal molars and/or a loss of M_3_. Although the correlation coefficient *r* was not high between individuals (Table 2), individual variation reflects a large number of environmental factors. Therefore, a significant correlation indicates that individual variation reflects an inhibitory cascade. Individual variation indicates the variability within a species (Klingenberg 2005); therefore, canid species differ from murine rodents in terms of variability, that is, they have lower slopes (Table 2). These patterns of variability are likely to be the source of the unique interspecific variation observed in canids.

**Figure 3:**
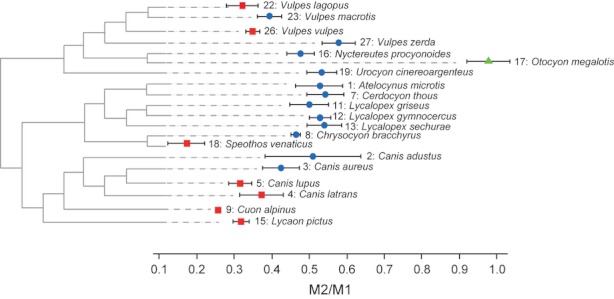
M_2_/M_1_ scores for each species, and their phylogenetic relationships from Bardeleben et al. (2005). Colors and figures indicate diet as in Fig. 1. Numbers indicate species as in Table 1.

The results of interspecific and individual variation in M_3_ loss indicate that M_3_ loss in canids must be generated by greater inhibition during evolution. Individual plots for *V. lagopus* provided a good illustration of interspecific variation; *V. lagopus* individuals with missing M_3_ were plotted near *C. alpinus,* a species that has lost M_3_, whereas individuals with normal dentition were plotted near species that retain M_3_ (e.g. *Vulpes vulpes*, *Vulpes macrotis*) (Figs. 1 and 2). Therefore, *V. lagopus* may be in a so-called ‘transitional stage’ of evolution of dental formulae, reflecting greater inhibition of the inhibitory cascade along the trajectory of carnivorous adaptation (Fig. 3). Nevertheless, the *N. procyonoides* population exhibited a high frequency of missing M_3_ despite relatively lower inhibition in relation to *V. lagopus* or other canids that retain M_3_ (Figs. 1 and 2). This *N. procyonoides* population has probably been affected by inbreeding depression, as the population examined was introduced from the mainland to a small island (Saeki 2009). The molar proportion of this population overlapped with that of the mainland populations that retain M_3_ (personal observation). Geographical isolation and fixation of series of genes that relate to the inhibitory cascade or other mechanisms could also be an important process in the evolution of dental formulae (Asahara et al. 2012). The fact that no *V. lagopus* and *N. procyonoides* individuals exhibited M_3_/M_1_ scores of lower than 0.03 and 0.05, respectively, may relate to additional mechanisms for the regulation of M_3_ development and a possible threshold for M_3_ development or loss; teeth germ which are smaller than some threshold at the critical stages cannot continue to develop into mature teeth (Gruneberg 1951; Wolsan 1989; Szuma 2003).

### Functional consequences of the unique inhibitory cascade pattern in canids contributing to the evolvability of diet

The patterns of interspecific variation shown here are indicative of a unique inhibitory cascade pattern with less steep regression lines in the morphospace (Fig. 1, Table 2) than any other previously reported mammals (Kavanagh et al. 2007; Renvoisé et al. 2009; Wilson et al. 2012). Guided by this pattern, the change in relative size of M_1_ has been amplified in canids (e.g. M_1_ comprises >80% of the total molar surface in *S. venaticus*, whereas the maximum proportion occupied by M_1_ in murine rodents is 66%; Fig. 1). My analysis is based on the two-dimensional occlusal surface, as used in previous studies (Kavanagh et al. 2007; Renvoisé et al. 2009; Wilson et al. 2012). However, the canid M_1_ is a high cuspid tooth in relation to other canid molars, or all molars of rodents; therefore, if analysis is based on the three-dimensional tooth volume, the change in the canid M_1_ must become further amplified.

Canids have evolved different proportions among functionally distinct parts of their lower molars, that is, the shearing surface (trigonid of M_1_), which is important for a carnivorous diet, and the grinding surface (talonid of M_1_, M_2_, and M_3_), which is important for omnivorous and insectivorous diets (Van Valkenburgh and Koepfli 1993; Friscia et al. 2007). Therefore, the particular pattern of inhibitory cascade (with amplified change in M_1_) would contribute to dramatic changes in the proportion of shearing surface in M_1_ versus grinding surface in M_2_ and M_3_ (i.e. dramatic changes in function) over the course of evolution. This dramatic change is regulated by a single developmental mechanism, the inhibitory cascade. Therefore, the molars of canids can readily evolve to adapt to a carnivorous, omnivorous, or insectivorous diet, and canids thus have the potential for evolutionary plasticity in their diet. These patterns of variation must have contributed to the diversity of dietary patterns and their parallel evolution among canids (Fig. 3) (Goswami 2010; Sillero-Zubiri 2010), and to the short-time divergence and diversity of dietary patterns in *Lycalopex* species (Perini et al. 2009). Polly (2007) inferred the existence of inhibitory cascade regulation across all mammals. In addition, previous studies (Renvoisé et al. 2009; Wilson et al. 2012), and my results, suggest that patterns of the inhibitory cascade can differ among taxa. Moreover, I suggest that these different inhibitory cascade patterns have contributed to different evolvability and diversity of diet among taxa. That is, clade-specific modification in developmental mechanisms could have promoted the capacity for dietary adaptation; that is, the dynamics of proximate and ultimate factors. Investigation of inhibitory cascade patterns in other mammals will further our understanding of these evolutionary dynamics.
